# Prosocial spending encourages happiness: A replication of the only experiment reported in Dunn, Aknin, and Norton (2008)

**DOI:** 10.1371/journal.pone.0272434

**Published:** 2022-09-07

**Authors:** Garam Kim, Ingrid Adams, Malik Diaw, Mira Celly, Leif D. Nelson, Minah H. Jung

**Affiliations:** 1 Department of Economics, New York University, New York City, New York, United States of America; 2 Marketing Department, Leonard N. Stern School of Business, New York City, New York, United States of America; 3 Marketing Department, Haas School of Business, University of California, Berkeley, United States of America; Iowa State University, UNITED STATES

## Abstract

Spending money on one’s self, whether to solve a problem, fulfill a need, or increase enjoyment, often heightens one’s sense of happiness. It is therefore both surprising and important that people can be even happier after spending money on someone else. We conducted a close replication of a key experiment from Dunn, Aknin, and Norton (2008) to verify and expand upon their findings. Participants were given money and randomly assigned to either spend it on themselves or on someone else. Although the original study (N = 46) found that the latter group was happier, when we used the same analysis in our replication (N = 133), we did not observe a significant difference. However, we report an additional analysis, focused on a more direct measure of happiness, that does show a significant effect in the direction of the original. Follow-up analyses shed new insights into people’s predictions about their own and others’ happiness and their actual happiness when spending money for themselves or others.

## Introduction

Human altruism helps to maintain and support the links between communities in society [1]. Because they seek to understand humans and their communities, economists and psychologists have tried to understand what leads to human altruism, and what are its consequences. Perhaps one of the most important and robust consequences of altruistic behavior is that it can bring happiness to the person who offers it [2]. It is not necessarily surprising that people are made happier by showing kindness to others, but it is quite surprising that being kind to others can bring more happiness than being kind to one’s self.

In a groundbreaking set of studies, Dunn et al. [3] demonstrated exactly that: people feel happier after spending on others than after spending on themselves. This finding has been highly influential, by changing how people think about the consequences of pro-social behavior, and by inspiring a number of follow-up investigations [4, 5].

The original paper collected evidence from a number of places, but the critical experimental results appeared in one study. In that experiment, participants were randomly assigned to either a personal or prosocial condition. In the personal condition, the experimenter instructed participants to spend money on themselves, whereas in the prosocial condition, the experimenter instructed participants to spend money on other people. Additionally, the researchers manipulated whether participants were given either $5 or $20 to spend, though this variable was shown to have no effect on the dependent variable. The critical dependent variable was self-reported happiness.

There are, however, reasons to think that the original evidence was imperfect. As was entirely the norm with other contemporary research, the original investigation was not pre-registered, had a relatively small sample size (n = 46 across four conditions), and had a critical statistical test that yielded *p* = .042, which may suggest limited evidential value. Specifically, Simonsohn, Nelson, & Simmons (2014) demonstrate that significant effects with p-values closer to 0.05 have less evidential value than significant effects with a lower p-value. The context is therefore ideal for a replication attempt: The original finding is undeniably important, but the evidence supporting it is undeniably imperfect. Any outcome would be informative.

We are not the only researchers to think so. Recently, the original authors and their colleagues conducted a registered replication of their original finding [6]. That paper reports three studies, all potentially informing the original research question. Results were somewhat mixed, but largely supportive. Nevertheless, the studies in the registered replication report are closer to conceptual replications than they are to direct replications. In Studies 2 and 3, for example, participants were asked to recall an experience in which they spent money on themselves or someone else, and then measured their well-being. In one of these two studies, the recall manipulation failed to reject the null, whereas in the other study, with a considerably larger sample size (N = 5,199), there was a small effect of prosocial spending. As is evident in this description, those studies are consistent with a small but real effect. Importantly, however, they meaningfully deviate from the original paradigm. It is not any less interesting, but thinking of a time when you spent money on someone else is quite different than being given money and being randomly assigned to spend it on someone else.

Study 1 of Aknin et al. [6] is the closest to the original experimental paradigm, used a considerably larger sample (N = 730), and generated statistically strong evidence (*p* < .001). In fact, the authors indicated that they improved the original paradigm in a few different ways. For example, unlike the original study in which participants were asked to spend $5 or $20 for themselves or others, in the replication study participants could buy a goody bag for themselves or for others, with identical items. This procedural change was intended to “[ensure] that any emotional differences observed across conditions are not a result of purchasing different content (e.g., experiences vs. material goods).” In addition, participants in the registered replication study did not directly interact with their recipients and their decisions were private such that any emotional outcome from giving cannot be fully explained by the recipients’ gratitude or compliments. Furthermore, and probably least consequential, the amount of giving was reduced to $2.50, rather than the $5 or $20 in the original. Finally, whereas the original had allowed participants to select their own recipients, the experiment in the replication report directed all prosocial spending towards sick children at a local children’s hospital. The changes are important to note, and may well assist in eliminating some potential confounding factors from the original. Of course, the meaning of those changes necessarily suggest a greater deviation from a direct replication. The replication experiment is not necessarily worse than the original, and is quite likely “better” in a number of ways, but it is not the most direct replication.

The results of Aknin et al. [6] are informative for the broad hypotheses about the benefits of pro-social spending, but they do not speak as directly to the necessarily narrower question as to whether the original finding would replicate. That is, it is still unclear whether prosocial spending increases happiness when people can freely choose for whom they want to spend and exactly how they choose to do so. The answer to this question can have broader application of prosocial spending in enhancing well-being beyond specific charitable giving contexts that Aknin et al. [6] tested. Therefore, we sought to answer that narrower question to probe the potential for broader application of prosocial spending to enhance well-being.

We conducted a close replication study of the original experiment reported in Dunn et al. [3] to test whether prosocial spending influenced well-being. We stayed close to the original paradigm (though we discuss deviations throughout), giving money to all participants and randomly assigning them to spend the money on themselves or on someone else.

Though our replication was intended to be a close replication, the replication was not exact. Some deviations stemmed from practicality whereas others were intended to extend the original finding collecting additional insights into the effect of prosocial spending. We next lay out the most salient differences between the original study and our close replication study. First, rather than manipulating the spending amount ($5 vs. $20 in the original), we chose a single value ($10) that fell between the two used in the original, and gave this amount to all participants. Because the original study showed that the size of the spending did not influence happiness, and our key interest the influence of spending recipient on happiness, we restrict our experiment only to a manipulation of that variable.

Second, we added an additional happiness measure to test whether the effect depended on how happiness was measured. The key analysis in the original study measured happiness with an 11-item measure that combined the Z-scored 10 positive emotion items from the PANAS [7] with one additional report of overall happiness at the end of the day. The critical specification in the original also controlled for a composite of those same measures, collected before participants were randomly assigned to one of the two conditions. We collected all of those measures and added a one-item happiness measure to test whether prosocial spending had a persisting effect. Specifically, after answering the 11-item happiness measure, participants in our replication study responded to the one-item measure, “how happy were you immediately after spending the $10 on yourself (someone else in the prosocial spending condition)?”

Third, we also added a pair of new questions to assess how participants forecasted the emotional consequences of spending. Specifically, prior to spending money, participants predicted their own happiness, and the happiness of their recipient, as a consequence of that spending.

The additional information afforded by these measures also raises a question as to whether their mere measurement influenced subsequent reports. Could this affective forecasting measure change self-reported happiness? Perhaps, for example, participants could feel pressure to show consistency between forecasts and later experiences. This is certainly possible, but would go against findings from the vast literature on affective forecasting. In those investigations, people often deviate considerably from their own forecasts even in a short-term lab paradigm [8, 9]. For example, in one paradigm, students were asked to predict how they would feel after receiving a test grade, and then immediately after receiving the grade reported how they felt. Although both measures were collected in the same setting, there were large and significant differences between forecasts and experiences [8]. Another study, reported in Mallet, Wilson, and Gilbert [9], participants forecasted their feelings immediately before a 5-minute conversation with a partner, and then immediately reported their feelings after. Once again, there were large differences between the two measures. Pressures for consistent responding were small enough not to block the measured effect, despite being measured very closely in time, and in the exact same setting. These findings suggest that instead of being consistent with their forecasts, people reliably divert from their forecasts that they made a short time before their actual experience. Even though the predicted and actual emotional responses tend to correlate, many affective forecasting studies still detect systematic errors at the mean levels of those responses, suggesting that the affective forecasting errors are not merely driven by the demand or consistency effect. Furthermore, people tend to misremember their forecasts to be consistent with their actual experience [10], suggesting that the consistency is biased toward the actual experience than toward the forecasts. In our study, participants came to the lab in the morning and were randomly assigned to the personal or prosocial spending task. They were asked to complete their spending task before 5pm that day. They were then told that they would receive a link to the second portion of the study before 8pm that day. Therefore, there were many hours in between the times when participants made their predictions in the morning and when they reported their actual happiness that evening, and the two measures were collected in entirely different settings. We predicted and observe similar correlations between predicted and actual happiness in our study (*r* (131) = .84, *p* < .001). But as we detail in the results section, we neither predicted nor observe a systematic forecasting error across the spending the conditions. We further discuss the possibility of the demand effect in the results and General Discussion sections.

Fourth, another difference between the original study and our study is the way key dependent measure was collected. In the original study in Dunn et al. [3], the researchers conducted a phone interview with the participants to record their emotional experience of the spending task. Therefore, participants might have been more engaged in the phone interview or the interaction with the experimenter over the phone induced a demand effect of its own on participants’ reporting of happiness. In our study, participants received an email with a link to report their experience.

Finally, we also modified the study instructions for clarity, as detailed in the procedure below. There were other differences as well, but they were largely beyond our control. For example, the original paper was published in 2008, and our study was conducted in 2018. The original was conducted at a large public Canadian university with students who were approached by an experimenter on campus (University of British Columbia), whereas ours was conducted at a large US university (University of California, Berkeley), with students enrolled in a participant pool. Exact replications are hard or impossible to conduct, but our hope was that ours was close enough to be informative for the original hypothesis. We also report some relevant exploratory analyses. The method section details our procedure, preregistration (https://aspredicted.org/tj87d.pdf) and the exact materials and data are all posted at http://researchbox.org/784&PEER_REVIEW_passcode=ILSNSC.

## Method

### Participants and design

To comment on the replicability of the original effect size, it has been recommended to collect a sample 2.5 times the size of the original [11]. By that standard, we targeted a sample of at least 115 participants and ended up with a sample of 137 participants. Undergraduates enrolled in an introductory marketing class at the University of California, Berkeley received partial course credit for participation. All participants were randomly assigned to one of two conditions: Personal (*n* = 67) or Prosocial condition (*n* = 66). Four participants failed to complete the entire study and, per our preregistration, were excluded from our analysis. Our final analyses, therefore, includes a total of 133 participants. There were three participants who completed the study but failed to follow instructions accurately: two participants instructed to spend the money on themselves instead spent the money on someone else, and one participant instructed to spend the money on someone else instead spent the money on themselves. These exclusions change neither the direction nor the statistical significance of the primary results. The relevant institutional review boards of the authors’ universities approved the study reported in this research. We obtained participants’ consent online using Qualtrics, an online data collection tool licensed for use in academic-related research. Participations’ consent and responses were administered and recorded through the survey platform. Participants were able to proceed to complete the study only after they consented to participate.

### Procedure

#### Pre-spending survey and assignment to condition

Unless otherwise specified, all procedures closely followed those of Dunn et al. [3]. Participants completed all survey items while seated in individual cubicles. The survey items included the Positive and Negative Affect Schedule (PANAS) and a measure of *baseline happiness* (“what is your overall happiness today?”) on a 5-point scale anchored at 1 (“not happy at all”) and 5 (“extremely happy”).

All participants were then told that they would receive $10 to spend after they leave the lab and before 5pm on that day. Participants in the personal condition were told, “we would like you to spend $10 on yourself today before 5pm. You can spend the money on a gift for yourself.” Note that in the original experiment, the materials suggested that participants “spend their money on a bill, an expense, or on a gift for themselves.’’ Participants in the prosocial condition were told “we would like you spend $10 on someone else today before 5pm. You can spend the money on a gift for someone else.” In the original study, those in the prosocial spending were told to “spend on a gift for someone else or charitable donation.” We slightly modified the original manipulation mainly to maximize the comparability of the conditions in the two spending conditions; the only difference was the target recipient being themselves or someone else. In considering the original instructions, we worried that spending on a bill or an expense for oneself might feel qualitatively different from spending on a gift for oneself. Similarly, spending for someone else might be qualitatively different from donating to a charitable organization that typically gives to those who are in need. Therefore, we simplified the manipulation with “spending on a gift for” oneself or someone else. In essence, we adjusted the spending instructions to remove an appeal to spend on charity in the prosocial condition and an appeal to pay off bills in the self-payment condition. As we return to in the discussion, this difference could influence our estimate of the replicability of the original. However, such a distinction would effectively be contending that people enjoy electively giving to charities more than paying of required bills, a considerably less broadly inclusive idea than the one that motivated the original and subsequent papers.

Finally, participants answered two questions that were not present in the original. Participants answered a *pre-spending happiness* question, “how happy will you be immediately after spending $10 on yourself” in the personal spending condition or “how happy will you be immediately after spending $10 on someone else,” in the prosocial spending condition on a 5-point scale. In the prosocial condition, participants also reported how happy they thought the recipient would be.

At the end of the survey, all participants were given the envelope of $10 and asked to complete their spending task by 5 pm on the same day. Participants received an email around 5pm with a link to the post-survey and asked to complete it by 8 pm that day.

In the post-survey, participants again completed the same PANAS and baseline happiness measure. They then answered a manipulation check question asking whether or not they spent $10 they received earlier that day. They then indicated *post-spending happiness* (“How happy were you immediately after spending the $10 on yourself (vs. someone else)”) on the same 5-point scale. They also indicated the amount of money they spent (“how much did you pay when you bought something for yourself (vs. someone else) today.” Both groups then answered a *counterfactual happiness* question asking how happy they would have been had they been assigned to the other condition. Specifically, in the prosocial spending condition, they answered, “How happy would you have felt if you spend $10 on yourself (a gift for yourself) instead of spending it on someone else?” In the personal spending condition, they answered, “How happy would you have felt if you spent $10 on a gift for someone else instead of spending on yourself?” Participants in the prosocial condition then predicted the recipient’s happiness (“how happy do you think the recipient of your gift of $10 was”). Finally, participants rated “the extent to which they had followed their assigned spending guidelines” on a scale with response options anchored at 1 (“not at all”) and 5 (“completely”) and were asked to explain how they spent the $10.

## Results

### Manipulation check

In the personal spending condition, participants typically bought a food or drink item (e.g., “I spent the money on coffee and pastries.”) or a small clothing or accessory item (e.g., “I spent it on a necklace in Brandy Melville.”). Similarly, in the prosocial condition, participants bought a food or drink item (e.g., “I bought a box of cookies for my friend.”) or a gift item (e.g., “got a scented jar candle). To probe whether our prosocial spending manipulation successfully induced participants to spend money for someone else or themselves, we asked workers on Amazon Mechanical Turk (N = 33) to code participants’ written descriptions of how they spent the $10. These coders were blind to the condition assigned to participants and rated the extent to which participants’ spending choices represented an expenditure for the (“self”) versus (“a gift for someone else”) anchored at 1 and 5 respectively on a continuous 5-point scale. On average, each coder rated about 33 randomly selected written descriptions. Coders exhibited extremely high consensus (Cronbach *α* = .87), rating participants in the prosocial condition higher (*M* = 4.03, *SD* = .80) than those in the personal spending condition (*M* = 1.99, *SD* = .70), *t*(128) = -15.52, *p* < .001, indicating the spending manipulation was successful.

### Primary analysis: Original specification used by Dunn et al. (2008)

As described above, Dunn et al. [3] measured happiness with a composite of 10 z-scored items from the PANAS along with a single 5-point scale of general subjective well-being. In addition, their analysis controlled for baseline happiness with a pre-test composite containing the same items (11-items averaged to be pre-windfall happiness). All dependent variables used in the original and this replication study can be found in Table 1.

**Table 1:** Happiness measures used as dependent measures and covariate in the original and replication studies.

		Happiness Measures
11-items composing pre/post-windfall happiness^a^	1-item Baseline	Overall happiness measured on a 5-point scale Interested
	10-item positive PANAS	Alert Excited Inspired Strong Determined Attentive Enthusiastic Active Proud
1-item pre/post-spending happiness^b^		Happiness immediately before/after spending money measured on a 5-point scale

^a^ Each of the 11 items that compose pre/post-windfall happiness was standardized, combined, and averaged.

^b^ One-item pre/post-spending happiness was used in the replication study as an alternative dependent measure.

Using the specification of the original, there were no pretest differences in happiness (*M*_*personal* spending_ = —.01 vs. *M*_*prosocial spending*_ = .01), *F*(1, 131) = .020, *p* = .887, consistent with successful random assignment to condition. This measure was used as a covariate in the analysis of post-windfall happiness.

**Fig 1:**
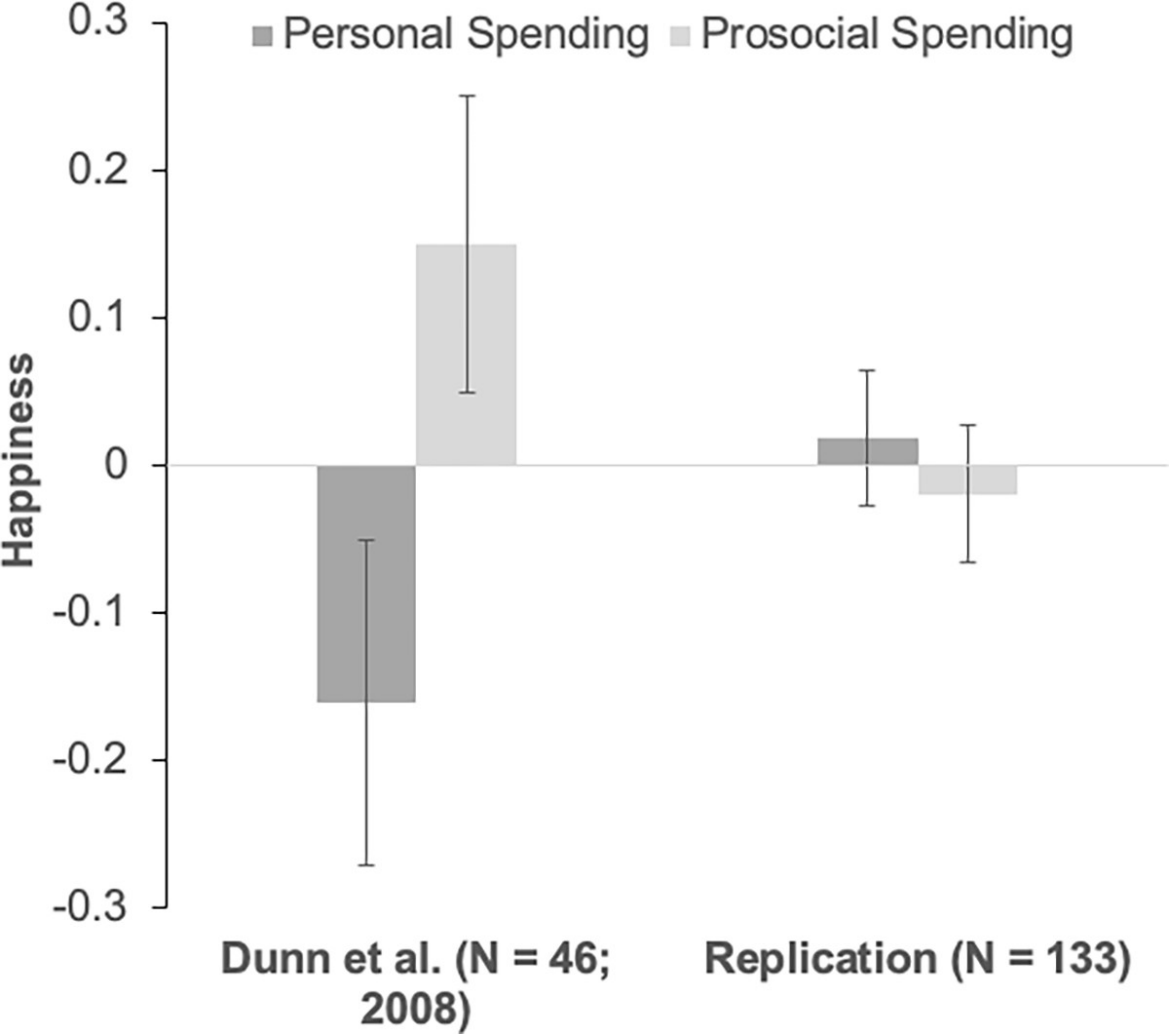
Post-windfall happiness from the two conditions. (A) Happiness reflects the post-windfall happiness, the 11 item composite (see Table 1 for the 11 items). (B) Error bars reflect ±1 standard error from the mean.

Again, following the analysis of the original, we analyzed post-windfall happiness while controlling for pre-windfall happiness. We first assessed whether including pre-windfall happiness as a covariate is appropriate. More specifically, we regressed participants’ post-spending happiness on their pre-windfall happiness, spending task, and the interaction term between the pre-windfall happiness and spending task. The results showed that participants’ pre-windfall happiness, *F*(1,129) = 11.12, *p* = .001, and the spending task, *F*(1,129) = 5.32, *p* = .023, were significant predictors of their post-spending happiness. But the interaction term between pre-windfall and spending task, *F*(1,129) = .43, *p* = .511, did not reach significance. Therefore, because including the additional interaction term left the critical main effect of prosocial spending largely unchanged, (*p*_*with interaction*_ = .023, *p*_*without interaction*_ = .022) including pre-windfall happiness as a covariate in the primary analysis was deemed appropriate. People who spent money on others were directionally, but non-significantly, less happy than those who spent money on themselves, *F*(1,130) = .35, *p* = .557, η_p_^2^ = .001 (see Fig 1). The result did not change when we excluded the pre-windfall happiness covariate, *F*(1, 131) = .04, *p* = .842, η_p_^2^ < .001.

We can use those numbers to estimate the power of the original study. The observed effect size has a 90% confidence interval to ranges from η_p_^2^ = .037 in the direction opposite of the original to η_p_^2^ = .009 in the same direction of the original. We can take the latter as our estimate of the upper bound of the true effect and then ask what was the upper bound for the statistical power of the original. The original study, with a total sample of 46 participants, is estimated to have slightly under 10% power to detect a significant effect. By this logic, although we cannot accept the null hypothesis, we can conclude that the original study was substantially underpowered to detect the alternative.

### Alternative measure of happiness

So, using the original measures and specification of the original, we did not observe a significant effect. Nevertheless, as we describe above, we collected an additional, more direct measure of happiness which asked people, simply, “how happy were you immediately after spending the $10 on yourself [vs. someone else].” In concept, this narrower measure might give a sense of change in happiness that was specific to the spending itself, rather than one integrated with every other contributing factor in someone’s life. An exploratory analysis using only this 1-item *post-spending happiness* did show a significant effect, with prosocial spenders being happier than personal spenders (*M*_*personal* spending_ = 4.04 vs. *M*_*prosocial spending*_ = 4.36), *t*(131) = 2.27, *p* = .025. A subsequent analysis, controlling for pre-windfall happiness (the 11-item composite), showed a similar result, *F*(1,130) = 5.34, *p* = .022, η_p_^2^ = .039. As described above, our best estimate is that Dunn et al. [3] had a maximum of 10% power to detect the hypothesized effect. Given differences between the original composite measure and our exploratory one item happiness measure, we do not directly compare the effect sizes from the two analyses.

In summary, in our replication attempt, the original specification of the analysis returned a null finding, but an alternative exploratory specification showed some evidence that prosocial spending increases happiness.

### How well can people predict the consequences of spending on happiness?

Participants predicted how their happiness would change as a result of their spending, using the same narrowed questions discussed above. In the pre-windfall they we asked “How happy would you be immediately after spending $10 on [someone else/yourself]?”. And immediately after spending, people answered, “how happy were you immediately after spending $10 on [someone else/yourself]?”

Did they predict accurately? We conducted a 2 (happiness: predicted (pre-windfall happiness) vs. actual (post-windfall happiness) × 2 (spending: personal vs. prosocial) mixed ANOVA with happiness as a within-subjects factor. Participants were slightly, but non-significantly, happier than they expected (*M*_*post-windfall happiness*_ = 4.20, *SD*_*post-windfall happiness*_ = 0.87 vs. *M*_*pre-windfall happiness*_ = 4.10, *SD*_*pre-windfall happiness*_ = 0.82), *F*(1, 131) = 2.90, *p* = .091, η_p_^2^ = .022. Furthermore, prosocial spending led to higher happiness reports (*M* = 4.31, *SD* = 0.76) than did personal spending, (*M* = 3.99, *SD* = 0.75), *F*(1, 131) = 5.90, *p* = .016, η_p_^2^ = .043. Notably, the interaction was not significant, *F*<1, suggesting that in this context, people were able to forecast the happiness consequences of the target of spending.

Could the mere inclusion of the forecasting question have influenced subsequent reported or experienced happiness? Perhaps, for example, the question induced a demand effect such that participants felt compelled to be consistent with their forecast. If true, then the pressure to be consistent should operate in both conditions.

#### Predictions about recipient happiness

We compared the pre- and post-spending happiness of those in the prosocial condition and their predictions about their recipients’ happiness. One participant in the prosocial condition was excluded for not answering the question and we included the rest in our analysis. Before spending money for their recipients, participants in the prosocial condition predicted that, after spending the money, their recipients would feel happier (*M* = 4.48, *SD* = 0.61) than they would themselves (*M* = 4.25, *SD* = 0.77), *t*(64) = -2.31, *p* = .024. Although all happiness estimates were slightly higher after the spending, participants still thought that recipients would be happier (*M* = 4.57, *SD* = 0.61) than they were themselves (*M* = 4.37, *SD* = 0.78), *t*(64) = -2.27, *p* = .027.

#### Actual happiness vs. counterfactual happiness

After completing the post-spending measures of happiness, all participants predicted their *counterfactual happiness;* how they would have felt had they been assigned to the other condition. We conducted a 2(happiness: counterfactual vs. actual (post-spending) × 2(spending: personal vs. prosocial) repeated measure ANOVA with happiness as a within-subjects factor. Overall, people thought that they would have been less happy in the alternative condition (*M* = 3.86, *SD* = 1.03), than in the one that they were assigned to (*M* = 4.20, *SD* = .82), *F* (1, 131) = 9.81, *p* = .002, η_p_^2^ = .070. There was no main effect of the spending condition, *F* (1, 131) = 1.49, *p* = .225, η_p_^2^ = .011. The interaction, however, was significant, *F* (1, 131) = 16.55, *p* < .001, η_p_^2^ = .112. Participants in the prosocial condition predicted that they would be less happy if they had been assigned to spend $10 for themselves instead for someone else, (*M*_*actual*_ *=* 4.36, *SD*_*actal*_ = .78; M_counterfactual_ = 3.56, *SD*_*counterfactual*_ = 1.01; *M*_*Difference*_ = .80, *S*.*D* = 1.30), *F* (1, 131) = 25.72, *p* < .001, η_p_^2^ = .164. But those in the personal condition thought that they would have been about equally happy to spend money for someone else, (*M*_*actual*_ = 4.04, *SD*_*actal*_ = .84; *M*_*counterfactua*l_ = 4.15, *SD*_*counterfactual*_ = .97; *M*_*Difference*_ = -.10, *S*.*D*. = 1.27), *F* <1.

## General discussion

We sought to replicate a key experiment from Dunn et al. [3] and observed mixed results. We did not replicate the result using the original measures and specifications. However, our exploratory measure, more narrowly focused on the experience of the spending itself, did find some support for the original analysis: People were happier after spending money on others than after spending money on themselves.

Additional analyses offered some additional insights that fit in with the literature of gift giving and pro-social spending. First, participants were largely accurate about the consequences of spending on their well-being. Second, prosocial spenders predicted that their recipient would be happier than themselves. Third, prosocial spenders thought that they would have been less happy if they had spent money on themselves, whereas personal spenders thought their happiness would not have been significantly higher had they spent on someone else.

Since we originally conducted our investigation, we have been in contact with the original authors, who have offered us insightful and even-handed feedback. They identified two critical issues. First, is an issue of statistical power. In essence, there are very good reasons to believe that our study was woefully underpowered to detect the effect under investigation, even if the effect is truly replicable. Those good reasons include appeals to both meta-analytic observation and naïve intuition. On the former point, Curry et al. [12] have suggested that positive psychology interventions have small-to-medium sized effects, requiring more than 200 observations per treatment to observe 80% power. The original investigation (N = 46) and this replication (N = 137) fall well short of that standard. If the Curry et al. suggestion is taken literally, a replication failure should not be all that surprising, since our investigation was considerably underpowered relative to that standard. By the same standard, of course, the success of the original should be seen as even more surprising. Still, though the increase in sample size from the original may not be enough to achieve 80% power by the standards of Curry et al., it is enough to comment on the power of the original investigation. In essence, if the current replication is judged to have sufficient fidelity to the original, then the replication cannot rule out whether the pro-social spending effect is true, but it can rule out whether the Dunn et al.’s sample was sufficient to detect it.

Is that enough? As the field of experimental psychology advances its focus on methodological rigor and statistical power, perhaps replications should aim to do more than merely consider the sample sizes of the studies they are investigating. The question boils down to a consideration of the goals or limitations of a replication study. Our sense, and indeed the way that we have interpreted our results, is that a replication can only comment on the specific design and evidence of the original investigation, and with that narrow focus in mind, the replication can only comment on the statistical power of the original. A different researcher might pursue a different goal and answer, perhaps, the question of whether the effect under investigation was truly that estimated in the Curry et al. meta-analysis. In concept, that could be fine as well, but it rapidly moves away from the clarity or simplicity of the narrow focus on the statistical power of the original. If, for example, the meta-analysis had suggested a study needs, say, 50 observations, and the replication used that as a guide, readers would be justifiably concerned that the replication lacked sufficient precision to comment on the original. That concern would be true at every level of the meta-analytic prescription. Alternatively, even if the meta-analysis suggests a sample of 200, a reader could (and absolutely should) wonder whether that meta-analytic estimate reflects an average of very different studies. Perhaps, for example, the target study was simply a more sensitive design than those included in the meta-analysis; it would be a disservice to the original to lump it in with other, less sensitive, designs. A replication is inherently indifferent to the average effect across a number of heterogeneous studies; it can only comment on the particular design and evidence from the original.

Of course, if the current replication is judged to have insufficient fidelity, then the assessment gets more complicated, which gets to the second issue raised by the original authors. They also noted that our new questions might have influenced the sensitivity of the old questions. Specifically, participants in our replication study were asked at the very beginning of the study to predict consequences of the spending for their own happiness, and that of their recipient. Perhaps, by merely measuring those constructs, we could have created expectancies which influenced either the experience or expression of happiness measured some hours later. It is an interesting possibility, and one that we do not have the data to rule out. On the one hand, expectancy effects can have wide ranging influences [13–15], and should not be discounted. On the other hand, the post-spending measure was collected at least many hours later and in a different setting; it would be interesting if such expectancy effects were still detectable.

Because we aimed to replicate the prosocial spending effect by staying close to the original paradigm, our replication study did not seek to address some potential shortcomings of the original study. A thoughtful reviewer suggested several possible explanations for the original prosocial spending effect on happiness. First, people might be buying different items for themselves (e.g., a sandwich) than for someone else (e.g., chocolates), and purchasing some items may bring more happiness than others. Although most participants in our study bought similar items for themselves as they did for someone else, any divergences could still be a potential source for an effect. Second, participants might have systematically selected people who were economically worse off than themselves as their recipients, and a comparable purchase might therefore be judged to have a larger relative influence. All participants in our study were college students in a public university and their recipients were often their peer students, suggesting relative economic homogeneity, but we do not have any direct evidence against the possibility that recipients were selected based on socioeconomic status. Notably, one of the conceptual replications by Aknin et al. [6] addressed part of this concern by fixing the gift items across all recipients. That procedural innovation meant that the gifts were held constant, but it also created a constant difference between givers, all of whom were undergraduate students, and their recipients, all of whom were sick children. Finally, the same reviewer asked, if people were happier to spend money for someone else as in the original study [3], and as Aknin et al. [6] showed, why do participants in dictator games typically offer 20–25%, not more than 50% of the pie with the recipients. This is a great question, and part of what makes the work by Aknin et al. [3, 6] so interesting. Stated more generally, if people want to maximize personal well-being, and pro-social spending brings more well-being than personal spending, why do we not see more pro-social spending? One possibility is that people cannot forecast that outcome, despite experiencing it. Our participants, for example, did not anticipate a well-being difference as a function of the recipient of their spending. Perhaps the surprise, therefore, is not that people fail to behave more generously (in dictator games or otherwise), because those people are merely acting in concert with their own imperfect theories of well-being. Rather, the surprise is that people have not learned the benefits of pro-social spending. It is at that point that the current paper could potentially mitigate some of the surprise with the least interesting outcome: perhaps pro-social spending does not in fact outstrip personal spending in influencing well-being. Though we are necessarily somewhat circumspect about the replicability of the original experiment reported in Aknin et al. [3], we are considerably more sanguine about the relationship more generally. In fact, even in our own data, we saw that people reported more happiness when asked about the spending specifically, even if that additional happiness was not detected in broader measures of general well-being. More generally, the positive consequences of prosocial spending might be most pronounced in certain situations (e.g., recipients are worse off than participants). Future research can examine the factors that reliably produce the prosocial spending effect such that one can better understand the mechanism and successfully apply the prosocial spending intervention to enhance well-being.

Prosocial behavior is important to understand for economists, psychologists, and policy makers. If pro-social behavior can truly generate more well-being for givers and receivers, then there is a tremendous opportunity for facilitating pro-social spending [16]. Although our replication was not very supportive of the original finding, it hardly obviates the original research question, and hopefully can contribute to ongoing investigations. Replication and open science allow for scientists to learn from results which are clearly supportive, clearly unsupportive, or perhaps ambiguously someplace in between.
